# Algorithm-improved high-speed and non-invasive confocal Raman imaging of 2D materials

**DOI:** 10.1093/nsr/nwz177

**Published:** 2019-11-13

**Authors:** Sachin Nair, Jun Gao, Qirong Yao, Michael H G Duits, Cees Otto, Frieder Mugele

**Affiliations:** 1 Physics of Complex Fluids, MESA+ Institute for Nanotechnology, University of Twente, Enschede 7500 AE, The Netherlands; 2 Medical Cell BioPhysics, TechMed Centre, University of Twente, Enschede 7500 AE, The Netherlands; 3 Physics of Interfaces and Nanomaterials, MESA+ Institute for Nanotechnology, University of Twente, Enschede 7500 AE, The Netherlands

**Keywords:** 2D materials, graphene, graphene oxide, confocal Raman microscopy, algorithm

## Abstract

Confocal Raman microscopy is important for characterizing 2D materials, but its low throughput significantly hinders its applications. For metastable materials such as graphene oxide (GO), the low throughput is aggravated by the requirement of extremely low laser dose to avoid sample damage. Here we introduce algorithm-improved confocal Raman microscopy (ai-CRM), which increases the Raman scanning rate by one to two orders of magnitude with respect to state-of-the-art works for a variety of 2D materials. Meanwhile, GO can be imaged at a laser dose that is two to three orders of magnitude lower than previously reported, such that laser-induced variations of the material properties can be avoided. ai-CRM also enables fast and spatially resolved quantitative analysis, and is readily extended to 3D mapping of composite materials. Since ai-CRM is based on general mathematical principles, it is cost-effective, facile to implement and universally applicable to other hyperspectral imaging methods.

## INTRODUCTION

Confocal Raman microscopy (CRM) has become one of the most widely used analytical methods to investigate the physico-chemical properties of 2D materials [1,2]. Compared to other methods such as optical microscopy [3–6], atomic force microscopy [3,7], and fluorescence quenching microscopy [8,9], Raman microscopy has the advantage to provide label-free, spatially resolved, compositional and structural information of the probed material on arbitrary substrates. Raman microscopy has enabled studies on the quality [10], defect [11], number of layers [10,12], crystal boundary [13], strain [5], oxidation state [14], and electron–phonon interactions [15] of 2D materials. For example, to determine the quality of fabricated graphene, it is common to present the characteristic Raman spectrum showing an intense *G′* peak (∼2700 cm^−1^), which is also referred to as the 2*D* peak (to avoid confusion with ‘two-dimensional’, we denote it as *G′*), together with a weak or absent *D* band (∼1350 cm^−1^) [10,16]. The quality can be further quantified by comparing the ratio of *G-* and *D*-band intensity [17]. For graphene oxide (GO), the changes in the intensity and peak position of the *D* and *G* (∼1600 cm^−1^) bands can be used to characterize its thermal reduction behavior [18]. For many other 2D materials such as boron nitride (BN) [19,20], transition metal dichalcogenides [2,21,22], and phosphorene [23], Raman microscopy has also become an important characterization tool.

However, the potential of Raman microscopy is severely hindered by its low throughput, due to the extremely low efficiency of the Raman scattering cross section: on average, 1 out of 10 million incident photons are Raman scattered. Typically, a single Raman spectrum of graphene takes hundreds of milliseconds to tens of seconds to acquire [24–30]. Consequently, a diffraction-limited map done across a 50 × 50 μm^2^ region, created by raster-scanning focal spots at a typical integration time of 1 s, would take half a day. As a result, many previous studies only collected single spectra from a few spots, despite the fact that large-area scanning and volumetric scanning are highly desired for accurate and systematic studies of material properties. In principle, the Raman signal intensity per pixel is proportional to the light dose, which is the product of the laser power and measurement time per pixel (and therefore inversely proportional to the scanning rate). A better signal-to-noise ratio (SNR) could be achieved by increasing the laser power. However, the laser power cannot generally be increased without considering potential light-induced damage to the sample. For graphene, structural changes are observed in response to laser irradiation in the mW range [31,32]. To mitigate this problem, an electron-multiplying charge-coupled device (EMCCD), available in many modern Raman instruments, has been used to amplify the SNR such that a higher scanning speed of several tens of milliseconds per spectrum can be delivered at 1 mW laser power [30,33]. Higher speed, however, is still needed for large-scale volumetric scanning. Recently, wide-field Raman imaging was introduced to map large-area graphene sheets in a few seconds [34]. However, this technique requires additional hardware components and is typically limited to one or a few frequency bands, undermining its performance for quantitative characterization of many physico-chemical properties such as defect density. The applicability in depth-resolved volumetric 3D scanning also remains challenging. For GO, the low throughput problem is even more severe due to the requirement of an extremely low laser dose to suppress reduction. A recent study suggested that even a laser dose of 48 μJ, i.e. 48 μW in 1 s, is still too high to prevent sample damage [35].

In recent decades, numerous studies demonstrated that imaging-related challenges can often be addressed with post-data-analysis using classical or modern algorithms [36–38]. Principal component analysis (PCA), an algorithm that is widely used in signal processing and machine learning to find common features in the dataset [39], has been applied to improve the SNR of hyperspectral datasets [40,41]. The idea is that, by analyzing the variance between spectra within the whole dataset, PCA can distinguish signal features from noise features and thereby allow a reconstruction of the dataset with predominantly signal features. The performance of PCA-guided denoising generally increases with the size of the dataset, because larger datasets enable a more thorough extraction of the signal features. Therefore, it is ideal for large-scale hyperspectral analysis.

In this work, we introduce algorithm-improved confocal Raman microscopy (ai-CRM), combining PCA and EMCCD, to image 2D materials. Briefly, we first collect spectra with an EMCCD at high scanning speed and low laser power. The combination of short measurement time per pixel and low laser power results in ‘noisy’ spectra with an SNR below one. Then we recover the faint signal, invisible in the noise, with the help of PCA. With this technique, Raman mapping of GO can be performed with an extremely low laser power of 5 μW, close to the hardware limitation, together with short integration times of 10 ms per spectrum. Meanwhile, we demonstrate that such a low laser dose per spectrum can effectively prevent GO reduction. Graphene can be mapped at a hardware-limited scanning rate of 1 ms per spectrum at 1 mW laser power. For graphene and GO, the power-averaged scanning rate (scanning rate divided by power for fair comparison) in our work is more than one order of magnitude faster than state-of-the-art works. Finally, we demonstrate that ai-CRM can be extended to fast imaging of other 2D materials including MoS_2_, WS_2_, and BN, and fast volumetric imaging of composite materials.

## RESULTS AND DISCUSSION

### Fast mapping of GO

**Figure 1.**
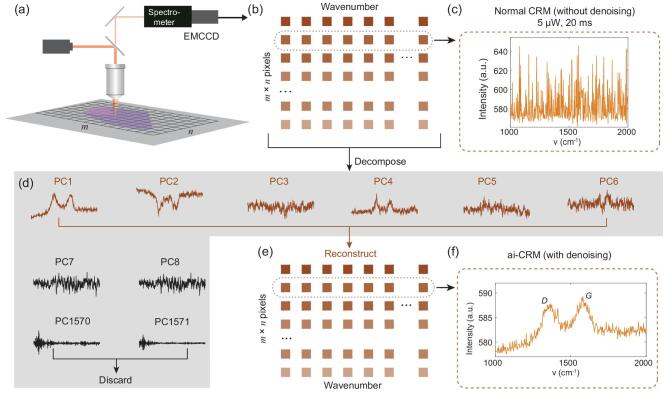
Schematic illustrating the ai-CRM procedure. (a) Raw dataset containing *m* × *n* pixels is collected using an EMCCD, and (b) assembled into a matrix in which each row contains a spectrum of a pixel and each column represents a wavenumber. (c) A representative noisy spectrum from a dataset recorded on a GO sample. (d, e) The PCs from a PCA of the measured dataset. PCA effectively decomposes the dataset into a low-dimensional space (here the first six PCs) containing mostly signal and a high-dimensional space (PCs seven to end) that contains mostly noise. PCs carrying signal information are used to reconstruct the dataset (e), whereas the rest of the PCs are set to ‘zero’ in the reconstruction. (f) Reconstructed spectrum of the same pixel as in (c) with dramatically improved SNR clearly showing the *D* and *G* bands of GO.

We first demonstrate our protocol (Fig. 1) with a typical Raman mapping of GO nano-sheets. Aqueous GO dispersion (1 mg/mL) was drop-cast on a plasma-cleaned 300-nm-SiO_2_/Si wafer. Confocal Raman mapping (Fig. 1a) was conducted by raster scanning over 25 × 25 μm^2^ with a step size of 0.25 μm, using a laser with a wavelength of *λ* = 532 nm. To avoid sample damage, the laser power was kept at a power of 5 μW underneath the objective. A 100 × objective with a numerical aperture (*NA*) of 0.9 was used, and the estimated laser spot size *d* is 1.22*λ*/*NA* = 0.72 μm, where *λ* is the laser wavelength, corresponding to a power density of 12.3 μW/μm^2^. The diffraction-limited resolution is *d*/2 = 0.36 μm. An EMCCD was used to collect the spectra with an integration time of 20 ms for each spectrum. After the scan, Raman spectra from all pixels were assembled into a data matrix where each row contained a spectrum (Fig. 1b). The Raman spectrum from 41 to 3692 cm^−1^ was selected for analysis. The collected spectra are extremely noisy, as shown by the representative spectrum in Fig. 1c. The Raman signal of GO cannot be identified. Subsequently, we applied PCA to decompose the dataset into its principal components (PCs; note that these components are not real spectra, but spectra of the variance in the dataset) whose number is equal to that of the number of wavenumber steps (1571 in this case). The PCs are ranked according to their percentage of total variance described, which represents the importance to the dataset [39]. Since the noise in the spectra is random, while signals are a recurring feature in a fraction of the pixels in the dataset, the PCs containing signals will contribute much more to the variance than those containing mostly noise (Supplementary Fig. 1). Typically, only the first few PCs contain obvious signal information. We use a scree test to determine the number of PCs (here, six) that need to be retained for further analysis (Supplementary Fig. 1) [42]. Relatively clear band-like features are observed in these first PCs (Fig. 1d), but not in the subsequent ones, which contain mostly noise. Using the first six PCs to reconstruct the dataset and rejecting 1565 ‘noise-dominated PCs’ out of the total of 1571 PCs, we obtain Raman spectra with dramatically improved SNR that clearly display the distinct *D* and *G* bands (Fig. 1f). The criteria to choose the number of PCs are loose. A few more or less than that suggested by the scree test is typically acceptable (Supplementary Fig. 2). The spectra in Fig. 1c and f correspond to the same pixel in the image.

**Figure 2.**
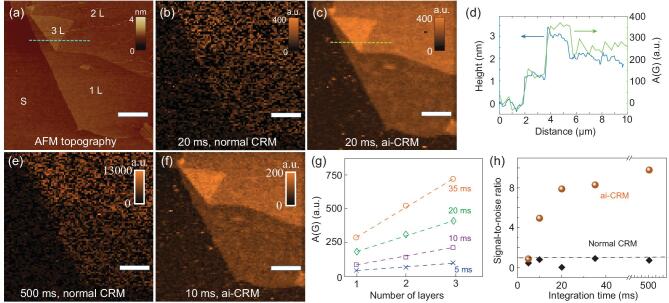
Fast Raman mapping of GO (*G* band) with ai-CRM. (a) AFM image of the probed GO sheets, showing the distribution of single layer (1 L), double layer (2 L), and triple layer (3 L) on a 300 nm SiO_2_/Si substrate (S). (b) Normal CRM raw image prior to denoising. At 20 ms integration time, the configuration of GO can only be vaguely discerned. (c) After denoising with ai-CRM, the configuration of the GO sheets can be clearly resolved, and (d) matches the AFM image in the pixel scale, as demonstrated by the largely overlapped AFM height profile and Raman intensity profile crossing the same positions (blue line in (a) and green line in (c)). (e) Normal CRM map with a high integration time of 500 ms. (f) ai-CRM image with integration time lowered to 10 ms, depicting the fastest mapping possible with our method without compromising on the layer identification. (g) Variation of the intensity with layer number for four different integration time, showing the possibility of fast quantification with ai-CRM. The dashed lines are guides to the eye. (h) Variation of SNR with integration time, depicting the amplification of the SNR achieved with ai-CRM as compared to normal CRM data. Scale bars are 5 μm and the resolution of the Raman maps are 100 × 100 pixels.

To demonstrate the efficiency of our method for mapping, we imaged the GO nano-sheet with an atomic force microscope (AFM) (Fig. 2a), and compared it with the Raman images (Fig. 2b and c) created by integrating the *G* band, with and without ai-CRM, respectively. For clarity, peak intensities will be denoted by *I* and integrated band area intensities will be denoted by *A*. The two images show the same region of interest. The distribution of single-layer (1 L), double-layer (2 L), and triple-layer (3 L) GO can be clearly resolved after applying ai-CRM (Fig. 2c). The integrated *G*-band intensity (*A(G)*) and AFM height profile on a selected line through the same positions (green line in Fig. 2b and blue line in Fig. 2a) shown in Fig. 2d demonstrates an excellent correspondence. In contrast, with normal CRM (i.e. without denoising), the GO nano-sheet can only be vaguely observed in the Raman intensity profile of Fig. 2b and the number of layers cannot be distinguished, even when the integration time per pixel is increased to 500 ms (Fig. 2e). Using ai-CRM, we can reduce the integration time even further down to 10 ms per pixel: the GO layers in the nano-sheet (Fig. 2f) are still clearly visible, confirming the efficiency of our method to facilitate fast mapping.

The ai-CRM data also allow quantitative analysis of the properties of GO. For example, the results (Fig. 2g) show that the integral intensity of the *G* band is linearly related to the number of layers and the integration time, which ranges here from 5 to 35 ms. This stems from the fact that the Raman intensity linearly scales with the volume of material within the confocal laser spot. This also implies that the 2 L and 3 L GO here are weakly coupled, suggesting that ai-CRM could be used for fast counting of layer numbers in the current system.

To quantify the SNR enhancement by our method, the average and the standard deviation of 400 randomly selected spectra were calculated for both normal CRM and ai-CRM datasets for each integration time. The SNR was then determined from:

(1) \begin{equation*}{\rm SNR}\ \, = \,\,I\!\left(G \right)/\Delta {I_\mathit{noise}},\end{equation*}

where *I(G)* is the peak intensity of the *G* band in the averaged spectrum and *ΔI*_noise_ is the standard variation calculated from the same spectrum at a wavenumber region (2000–2200 cm^−1^), which is a region where no bands of these materials occur. Figure 2h shows that with CRM, the SNR is lower than 1 for all integration times from 5 to 500 ms, leading to noisy images (Fig. 2b, e and Supplementary Fig. 3a). Note that an SNR below 1 implies that the signal cannot be clearly distinguished from noise, and therefore the values of SNR are only a rough estimation. With ai-CRM, however, the SNR increases dramatically and progressively increases with integration time. Importantly, even the SNR for 10 ms integration is much higher than for any of the integration times without denoising. This suggests that our method can increase the scanning speed by more than 50 times from 500 ms to 10 ms at an improved SNR. In addition, a literature review suggests that the power-averaged scanning rate (scanning rate divided by power for a fairer comparison since Raman signal intensity is proportional to dose) in our work is two to seven orders of magnitude higher than previous works (Supplementary Fig. 5a).

### Non-invasive mapping of GO

For GO, reliable Raman characterization has been a major challenge due to laser-induced sample damage [35,43]. To mitigate this problem, the laser dose needs to be reduced as much as possible [11,35,43]. Many previous works used mW scale power to characterize GO [44–48]. A recent study [35] suggested that laser-induced reduction of GO cannot be prevented even when the laser intensity is down to a dose of 8 × 10^7^ J/m^2^, which corresponds to 48 μW during 1 s in the confocal spot. Such laser intensity is already a few orders of magnitude lower compared to preceding studies [47,48]. Further reduction of the laser power, however, resulted in Raman spectra with insufficient SNR. However, the results in Fig. 2 suggest that ai-CRM enables characterization of GO at two orders of magnitude lower intensity (tens of ms with a laser power of 5 μW).

**Figure 3.**
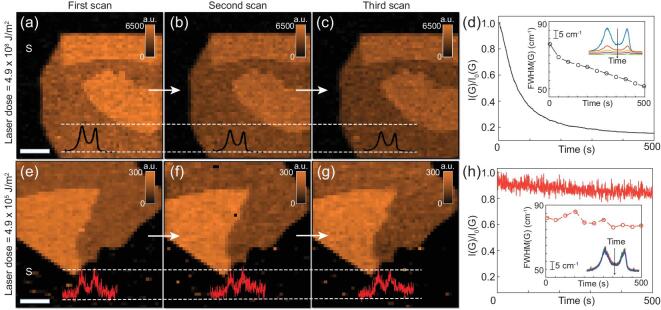
Reliable Raman mapping of GO at minimum laser power with ai-CRM. (a–c) Raman maps of GO (*A(G)*) done at the same location, one after the other using a CCD, with a laser power of 4 mW. (e–g) Raman images of GO done using ai-CRM, at 1000x reduced laser power (4 μW) showing minor loss of contrast and integrated intensity. (d, h) Variation of the normalized intensity of the *G* peak of GO, obtained from single-pixel time series data for 4 mW (d) and for 4 μW (h). The insets show the variation of the spectra as well as the FWHM of the *G* peak. All data recorded at 50 ms integration time per pixel. All scale bars correspond to 5 μm and all Raman maps have a resolution of 50 × 50 pixels.

For a comparative study with ai-CRM, we selected two levels of laser power, 4 μW and 4 mW (Fig. 3), to determine the influence of laser power on the Raman signal of GO. Note that for the 4 mW case, normal CCD instead of EMCCD was used, because at such high laser power in the EMCCD mode, the intensity of the silicon peak exceeds the upper detection limit. This has no effect on the sample damage analysis. Figure 3a–c shows the Raman images obtained at 4 mW of one nano-sheet of GO and Fig. 3d–f shows the Raman images at 4 μW of another nano-sheet of GO. All images in the same series are presented against the same color scale. Each pixel was measured for 50 ms in both situations. The loss in *A(G)* in going from Fig. 3a to Fig. 3c is apparent from the reduction of contrast at 4 mW (4.9 × 10^8^ J/m^2^ intensity), which indicates the gradual increase in sample damage upon repeated illumination. The decrease in Raman intensity is also seen in the average spectra for each image (insets in Fig. 3a–f). Laser-induced reduction (to reduced graphene oxide, rGO) during Raman imaging has been observed on all GO nano-sheets (see e.g. Supplementary Fig. 6). This was further confirmed by measuring Raman spectra of a single spot during 500 s of continuous laser irradiation of 4 mW with a spectrum obtained every 50 ms. The Raman intensity decreased rapidly by ∼ 70% in the first 100 s and gradually decreased further to a weak plateau value of residual scattering (Fig. 3d). In addition, the full width half maximum (FWHM) of the *G* band decreased monotonically with time (inset Fig. 3d), and the *I(D)/I(G)* ratio changes with time (Supplementary Fig. 6a), which is a typical consequence of the reduction of GO [35]. In contrast, the intensity loss is substantially reduced when illuminated with 4 μW of laser irradiation (4.9 × 10^5^ J/m^2^ intensity) for 500 s with 50 ms per spectrum (Fig. 3h and the insets, respectively). In addition, scanning did not cause any obvious optical changes to the bright-field image of the sample (Supplementary Fig. 6). After 500 s exposure at the same pixel, the change in *I(G)* is less than 10%. The change in *A(G)* during the illumination time per pixel in a Raman image is then less than 1 in 10 [5], which is negligible for most purposes. The change in FWHM is also negligible as it is close to the wavenumber resolution of the instrument (∼ 2 cm^−1^). These results clearly confirm the efficiency of ai-CRM to suppress sample damage after reduction of the experimental laser intensity.

### Fast mapping of graphene and its use in sample quality analysis

**Figure 4.**
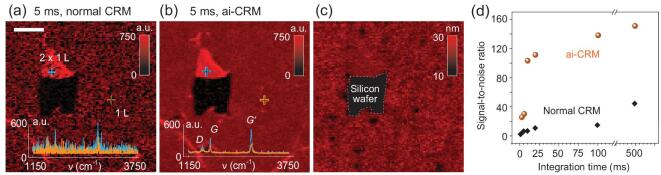
Fast Raman mapping and quality analysis of graphene with ai-CRM. (a) Normal CRM map of CVD-grown graphene on 300 nm-SiO_2_/Si wafer at 1 mW laser power and 5 ms integration time. The inset shows the spectra corresponding to the markers on a double single-layer (blue) and single-layer (yellow). (b) ai-CRM map of the same region. The spectra show the evolution of the *D*, *G* and *G′* peaks after denoising, useful for quantitative analysis. (c) Color-coded defect density map, derived from the peak intensity ratio of the *D* and *G* bands (Eq. 2), of the same region depicting that the average distance between defects is around 20 nm, showing the high quality of the CVD-grown graphene. (d) Variation of the SNR of the *G′* peak for various integration time, illustrating the amplification of the SNR with ai-CRM.

Our method can also be applied for fast Raman mapping of graphene. To demonstrate this, we made a scratch on a single layer of graphene grown by chemical vapor deposition (CVD) on a 300-nm-SiO_2_/Si wafer. Then we performed a Raman scan across 25 × 25 μm^2^ with a step size of 0.25 μm at 1 mW laser power, which was previously confirmed to be non-destructive for graphene [33]. Similar to the results of GO, significant SNR improvement was observed, as shown by the Raman images of the integral *G′-*band intensity generated at 5 ms integration time before and after applying ai-CRM (Fig. 4a and b). Before denoising, the *D*, *G*, and *G′* bands can hardly be seen (bottom spectrum in Fig. 4a). Although different layer numbers can still be distinguished, an accurate quantitative analysis is hardly possible. After denoising, a weak *D* band, a sharp *G* band, together with a sharp and strong *G′* band are clearly resolved (bottom spectrum in Fig. 4b), indicating the high quality of the graphene sample (refer to Supplementary Fig. 4 for mapping at other integration times using ai-CRM). The Raman intensity (*I(G’), I(G)* and *I(D)*) of the double-layer graphene (labeled as 2 × 1 L in Fig. 4a) is roughly twice that of the single-layer graphene, confirming that the double layer is composed of two weakly coupled single layers.

Importantly, the ai-CRM data enable quantitative analysis of the sample quality. The distance between defects, *L*_D_, can be estimated from the ratio of *I(G)* to *I(D)* [17]:

(2) \begin{equation*}L_{\rm{D}}^2\left( {{\rm{n}}{{\rm{m}}^2}} \right)\,\, = \,\,1.8{\rm{\ }} \times {10^{ - 9}}{\lambda ^4}I\!\!\left(G \right)/I\!\!\left( D \right)\!,\end{equation*}

where *λ* is in nanometers. The distribution of *L*_D_ is then plotted in Fig. 4c. It is observed that the graphene sample has a relatively uniform *L*_D_ of ∼20 nm across the scanned area, again confirming its high quality. Note that regions having *L*_D_ < 10 nm are not plotted (the scale bar in Fig. 4c starts from 10 nm) because Eq. (2) is not valid in this case. Note that the reduction of GO is often also quantified in terms of a reduction of the density of oxygen-containing defects in an otherwise perfect graphene lattice. The corresponding change in the *I*(*D*)/*I*(*G*) ratio is shown in Fig. 3d. The above results suggest the potential of ai-CRM for spatially resolved and fast characterization of the graphene quality, which should help the quality control of graphene fabrication.

Furthermore, to quantify the improvement in scanning rate, we scanned the same sample using different integration times from 1 ms to 500 ms, and calculated the SNR for all datasets before and after applying ai-CRM. The results in Fig. 4d show that the SNR at 500 ms without denoising is comparable to the SNR at 5 ms with denoising, and is much lower than that at 10 ms with denoising. Similar to the results for GO, this demonstrates again that an increase of more than 50 times in scanning rate can be achieved when ai-CRM is applied. Compared to the literature, the power-averaged scanning rate here is around two orders of magnitude higher than state-of-the-art works (Supplementary Fig. 5b).

### Fast mapping of other 2D materials

**Figure 5.**
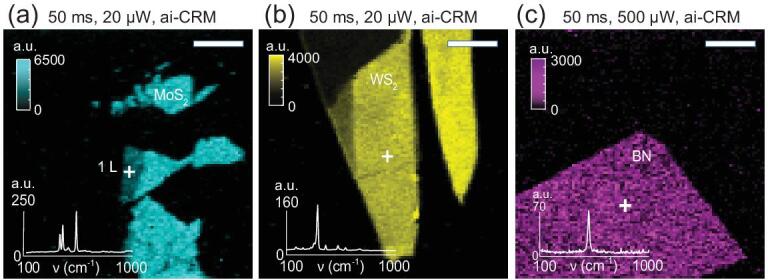
Fast mapping of mechanically exfoliated MoS_2_, WS_2_, and BN with ai-CRM. Raman mapping of (a) MoS_2_ (1 L at crosshair), (b) WS_2_ (∼5 nm at crosshair) and (c) BN (∼9 nm at crosshair) with denoising. The denoised spectra are shown in the inset at the location of the crosshair. The laser power and integration time are mentioned in the figures and the scale bars correspond to 5 μm. The resolution of the Raman maps is 100 × 100 pixels.

ai-CRM was also tested on mechanically exfoliated MoS_2_, WS_2_, and BN nano-sheets (optical and AFM images in Supplementary Fig. 7). MoS_2_ and WS_2_ can be imaged at 50 ms integration time under 20 μW laser (Fig. 5a and b). BN, having a smaller Raman cross section, can be imaged at 50 ms integration time under 500 μW laser (Fig. 5c). Note that the ai-CRM maps and spectra of these materials generated at 50 ms integration time have similar or better quality than normal CRM maps and spectra at 500 ms integration time (compare Supplementary Fig. 8), suggesting that ai-CRM increases the scanning speed by at least 10 times, still with an improvement in SNR.

### Fast volumetric imaging of an rGO composite

**Figure 6.**
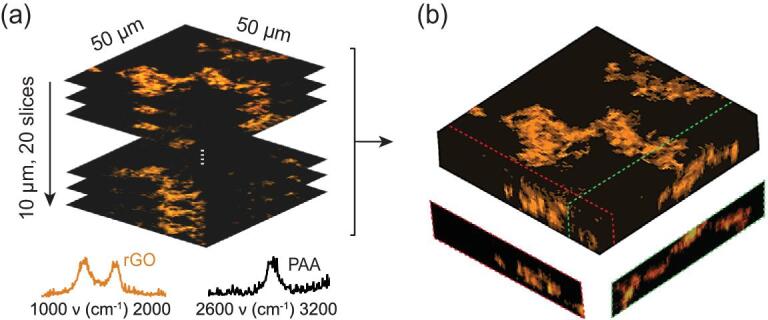
Volumetric 3D imaging of rGO-PAA composite using ai-CRM. (a) Denoised *x*–*y* Raman maps of rGO and PAA, taken with a step size of 0.5 μm. The laser power is 0.75 mW and the integration time is 1 ms. The spectra at the bottom correspond to rGO (orange) and PAA (black), respectively. (b) The 3D Raman reconstruction of the same nanocomposite, depicting the distribution of rGO in the polymer matrix. The orthogonal sections along the dashed lines show rGO embedded in the matrix. Total data acquisition time: 12 min.

Volumetric imaging is another advantage of CRM, which can help e.g. to assess the properties of graphenic materials inside a composite or a device. However, such a potential advantage has not been taken advantage of in previous studies, due to the required long measurement times. Since one 2D Raman image across tens of micrometers at diffraction-limited spatial resolution could take half a day, a volumetric Raman image created by stacked 2D images would take several days. With our method, it is now possible to reduce the time to around 10 min. To demonstrate this, a GO dispersion was mixed with aqueous polyacrylic acid (PAA), and the mixture was cured overnight in an oven at 80°C. After curing, GO is moderately reduced. Using ai-CRM with 0.75 mW laser power (a higher laser power is used because GO is already reduced) at 1 ms integration time, both rGO and PAA, respectively bottom left and right spectra in Fig. 6a, show their characteristic Raman spectra with the CH stretching band located at around 2930 cm^−1^ in the PAA spectrum. We subsequently scanned a vertical stack of 20 images across a depth of 10 μm. Each image in the stack covers 50 × 50 μm^2^ with a step size of 0.5 μm and 1 ms integration time (Fig. 6a). The imaging thus took 100 × 100 × 0.001 × 20 = 200 s pure measurement time, which took ∼720 s when the camera readout time and stage translation time were taken into account. Afterwards, the 3D distribution of rGO is plotted (Fig. 6b), showing how rGO is blended into the composite. Slices extracted from any arbitrary positions within the volumetric view can be visualized, as shown by the two cross section images in the bottom insets of Fig. 6b whose positions are labeled by the dashed green and blue lines.

### Fast mapping of graphene and GO on arbitrary substrates

Compared to other characterization methods such as fluorescence quenching microscopy and bright-field optical microscopy, CRM has the advantage of universal applicability on arbitrary substrates. As an example, we used ai-CRM to image a CVD-grown graphene on a glass substrate at 20 ms integration time with 1 mW laser power across 25 × 25 μm^2^ with a step size of 0.25 μm (Supplementary Fig. 9). GO can also be imaged on glass or calcium fluoride substrate with low laser power (10 μW) and short integration time (20 ms), as shown in Supplementary Fig. 9. The wrinkles and folds on the GO sheets are clearly visible.

## CONCLUSION

In this work, we demonstrate that ai-CRM significantly improves the SNR of the Raman spectra of various 2D nanomaterials such as graphene, GO, WS_2_, MoS_2_, and BN. Thereby, it increases scanning rates by more than 50 times with respect to conventional state-of-the-art CRM (Figs 2h and 4d). Introducing this improvement, sensitive samples such as GO can be mapped faster at extremely low laser powers of just several μW. This minimizes laser-induced sample damage and enables reliable and quantitative characterization of the physico-chemical properties of graphenic nano-sheets, such as layer number and defect density. Compared to other characterization tools, CRM has the advantages of providing label-free, substrate-independent, and 3D spatial information. Since the denoising performance increases with the size of the dataset, an even higher scanning rate is expected when the Raman mapping area further increases. This is a highly demanded property, because large-scale industrial production of 2D materials and their devices requires scalable characterization methods.

While other techniques such as surface-enhanced Raman spectroscopy [49] and stimulated Raman scattering [50] may give rise to even higher SNR improvement, these techniques are either technically much more involved or require specific substrates and samples and/or do not allow for volumetric imaging.

Since ai-CRM is based on a purely mathematical framework, it can also be applied to improve the above techniques instead of replacing them, and other hyperspectral microscopy methods, such as hyperspectral infrared microscopy and photo-luminescent microscopy. We therefore expect ai-CRM to strengthen the use of hyperspectral imaging as a fast, reliable, quantitative, and spatially resolved characterization tool in the fabrication and broad application of 2D materials.

## METHODS

### Materials

A GO suspension (2 mg/mL, 22 μm mean diameter flakes, Sigma Aldrich) was diluted with 1 mL of deionized water (Millipore) and was deposited on plasma-cleaned 300-nm-SiO_2_/Si wafers by drop-casting. CVD-grown single-layer graphene on glass and 300 nm SiO_2_/Si wafer was purchased (Graphene Supermarket, USA) and used directly for Raman imaging. Standard laboratory-grade glass coverslips and Raman-grade calcium fluoride (Crystran Ltd, UK) were used as alternative substrates. MoS_2_, WS_2_, and BN were mechanically exfoliated from their bulk crystals (HQ Graphene, the Netherlands) with Scotch tape. MoS_2_ and WS_2_ were directly exfoliated on 300-nm-SiO_2_/Si wafers. BN was exfoliated on polydimethylsiloxane film and then stamped on 300-nm-SiO_2_/Si wafer.

### Raman measurements

Raman measurements were carried out using a WiTec alpha 300R Raman microscope connected to a 532 nm laser. A 600 g/mm grating was used, which provided a spectral resolution of around 2.3 cm*^−^*^1^. An EMCCD camera (1600 × 200 pixels, 16 μm pixel size, Andor Newton) was used for detection of the scattered photons. The EMCCD gain was set at a numerical value of 250. The pre-amplifier gain value was set to 1. For high spatial as well as depth resolution, a 100 × objective (Zeiss EC ‘Epiplan-Neofluar’ DIC, numerical aperture (*NA*) = 0.9) was chosen. The laser power at the sample was measured using an optical power meter (ThorLabs). Raman maps are made by integrating the area under the band of interest for every pixel: from 1550 to 1750 cm*^−^*^1^ (*G* band) for GO and graphene, 2600–2800 cm*^−^*^1^ for the *G′* peak of graphene, 253–400 cm*^−^*^1^ for WS_2_, 1330–1390 cm*^−^*^1^ for BN, 350–427 cm*^−^*^1^ for MoS_2_ and 2800–3000 cm*^−^*^1^ for PAA.

### Denoising

Denoising was performed on MATLAB (version R2017b) with home-written codes without any pretreatment. A sample code and a sample dataset for graphene are given in the supporting information. Typically, denoising 10 thousand spectra only takes around 10–20 s with a normal office computer. After denoising, cosmic rays are also removed, because of their random nature.

## Supplementary Material

nwz177_Supplemental_File
