# Supplementary material for: Algorithm-improved high-speed and non-invasive confocal Raman imaging of 2D materials
Source: Natl Sci Rev. 2019 Nov 13;7(3):620–8. doi: 10.1093/nsr/nwz177 (PMC8289049; doi:10.1093/nsr/nwz177)
Supplement: nwz177_Supplemental_File [file nwz177_supplemental_file.docx]

**Supplementary Data for**

**Algorithm-improved high speed and non-invasive**

**confocal Raman imaging of two-dimensional materials**

**Selecting the number of components retained for PCA based denoising**

**
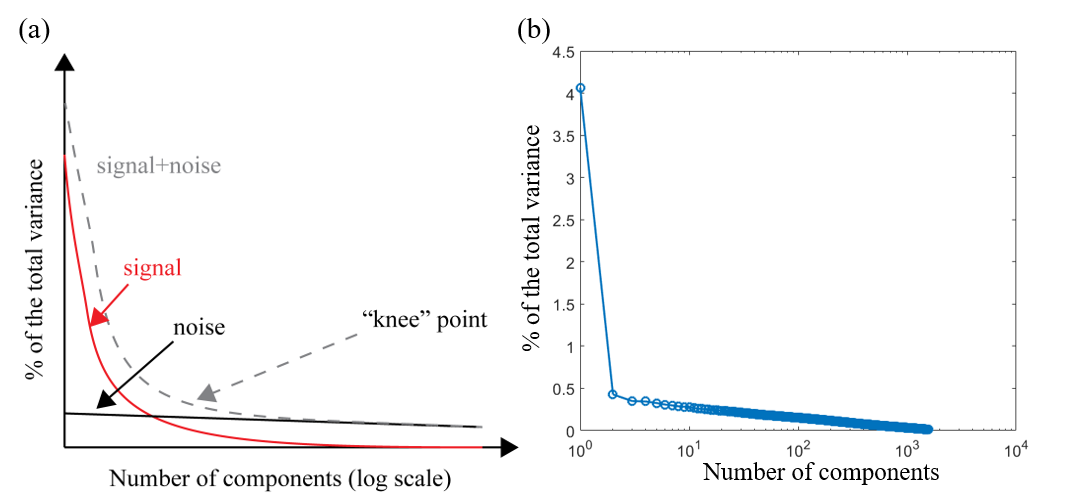
**

Supplementary Figure 1: **Mechanism of PCA guided denoising and Scree plot for Fig. 1 in the manuscript**. (a) PCA distinguishes signal and noise features by their different collective behaviors. Since noise is largely random, their contribution to the variance in different components are relatively constant and low. However, since signals are recurring features, their contribution to the variance will accumulate to a high value. PCA ranks the components according to variance, and therefore, the signals will be captured to the first few components. When the variance start to decrease slowly, mostly noise are captured. To select the “signal components”, *Scree test* is used. Briefly, the percentage of total variance (variance of each components divided by the total variance of all components) is plotted for all components. Then we find a “knee” point in the plot. Selecting a few more components at the right side of the knee point does not affect the denoising performance since most of the noise is rejected anyway. (b) Scree plot for Fig. 1 in the manuscript.

**Variation of the denoised spectra with the number of components used for reconstruction**

**
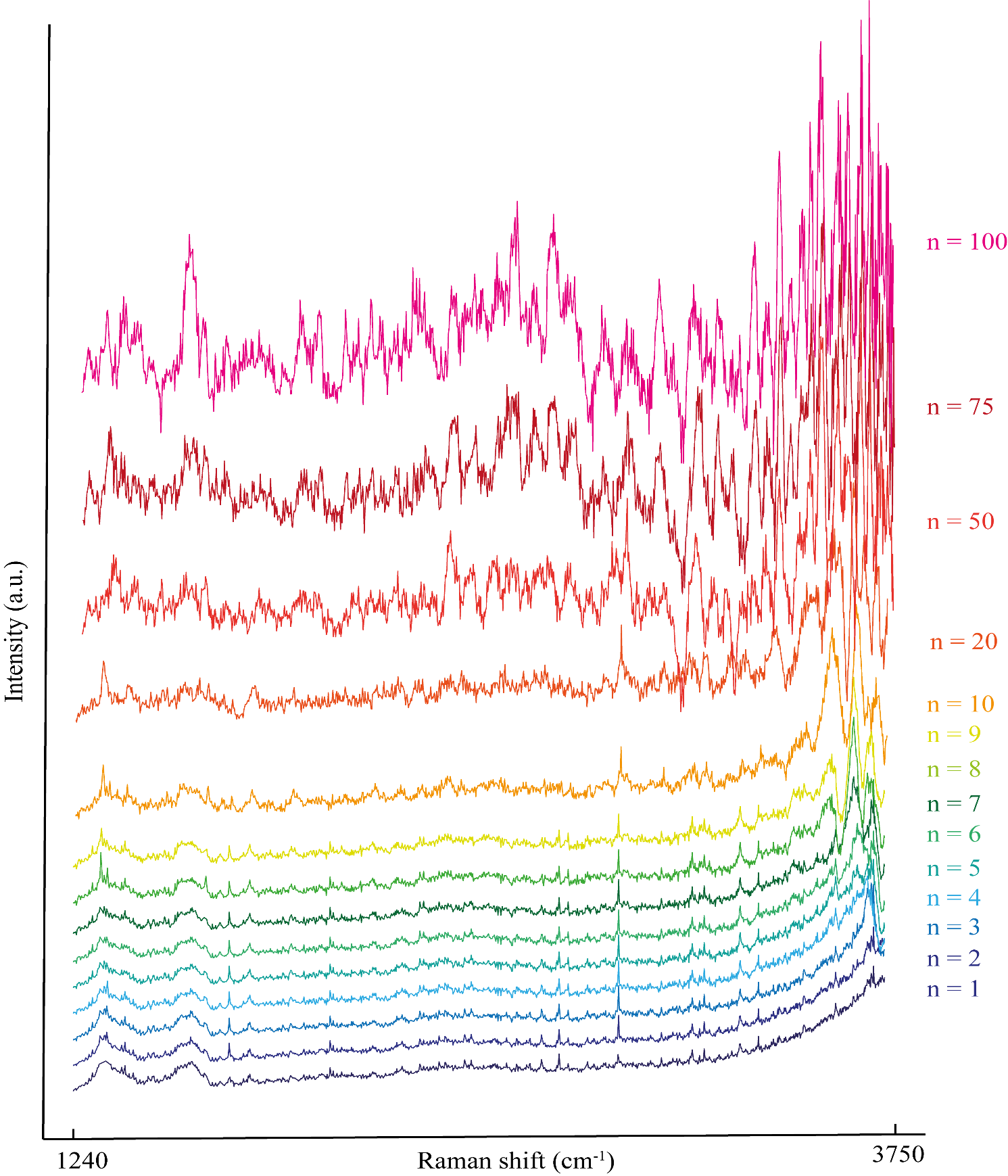
**

Supplementary Figure 2: **Dependence on the number of components retained corresponding to Figure 1(f) of the main text.** Evolution of the denoised single pixel spectra depending on the first *n* components used for reconstruction. The spectral SNR of the *D* and *G* peaks starts becoming worse, especially after *n*=10.

**Raman maps of GO and graphene at other integration times**


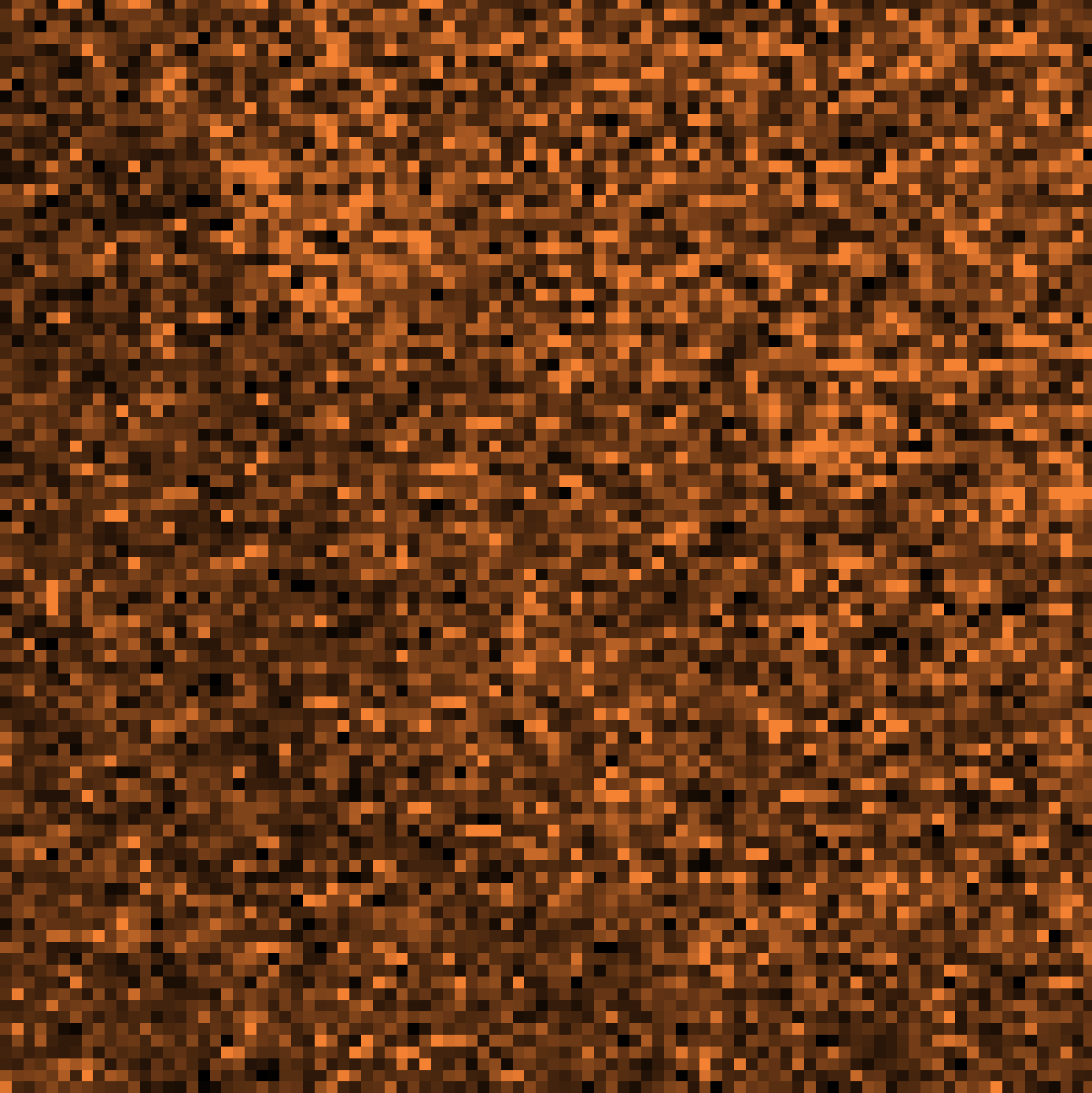

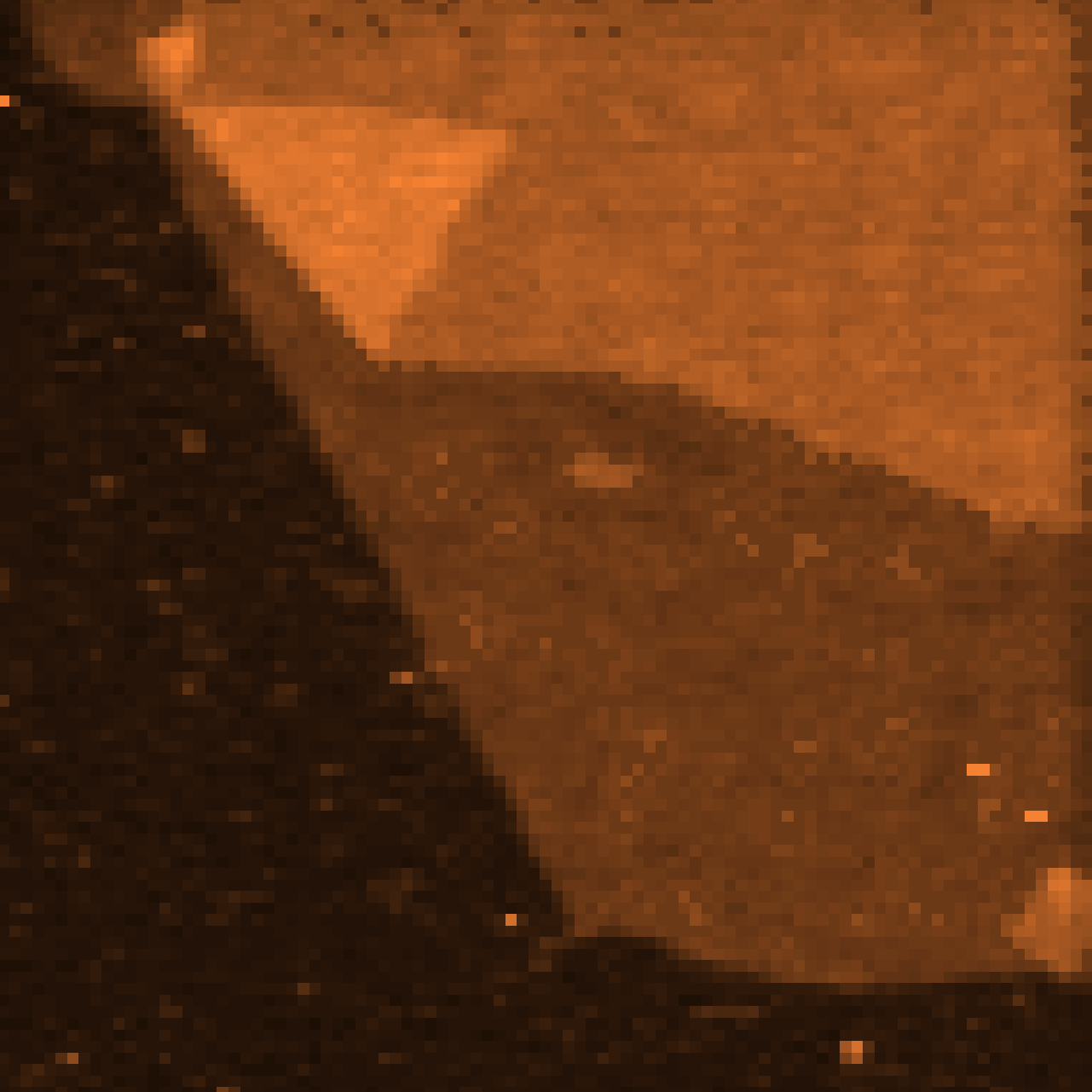

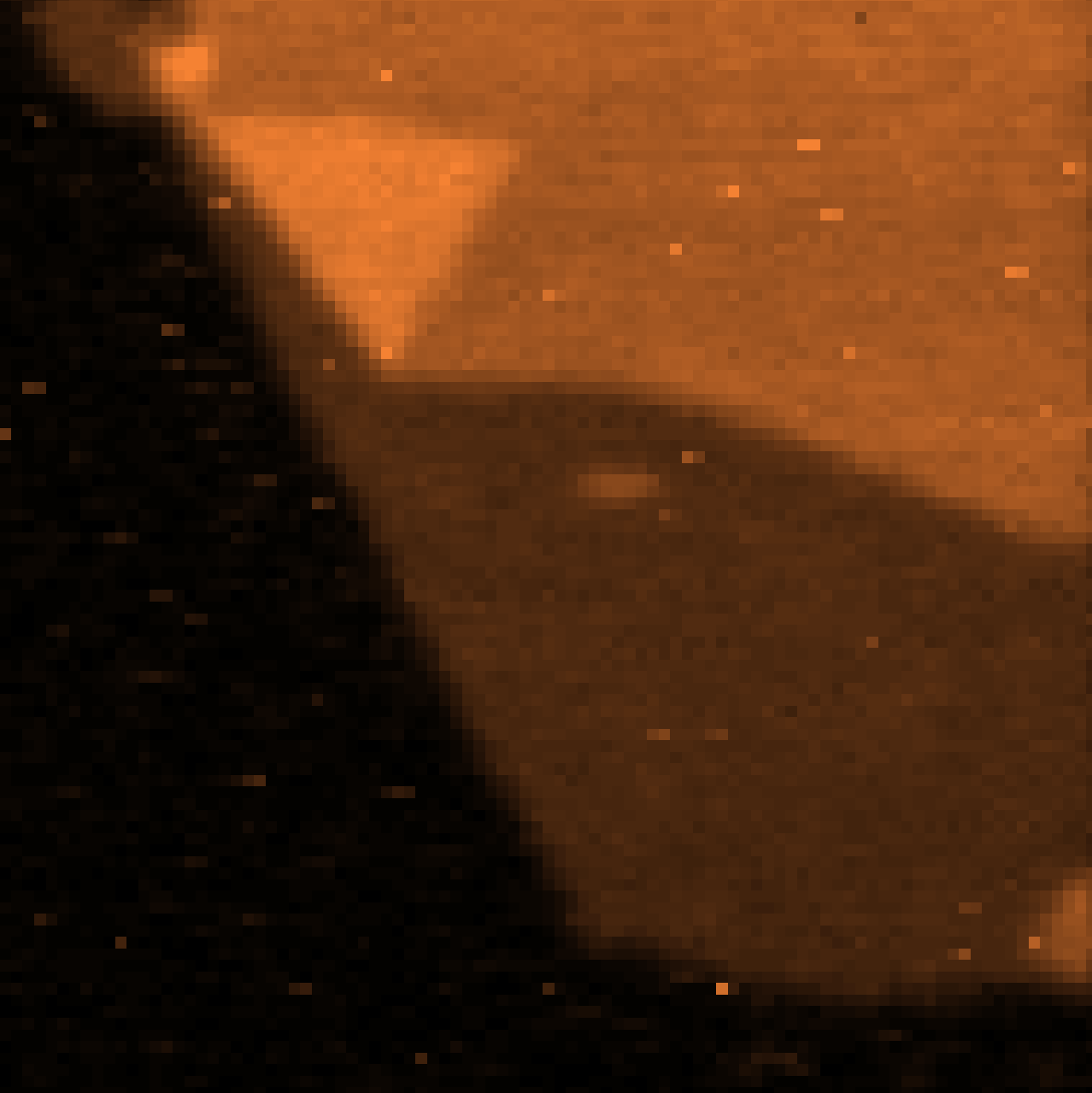

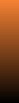


0

100

a.u.


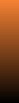


0

750

a.u.


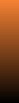


0

13000

a.u.

(a)

(c)

(b)

Supplementary Figure 3: **ai-CRM maps of GO corresponding to Figure 2 of the main text.** Denoised Raman maps at an integration time of (a) 5 ms, (b) 35 ms and (c) 500 ms. The scale bars correspond to 5 µm.


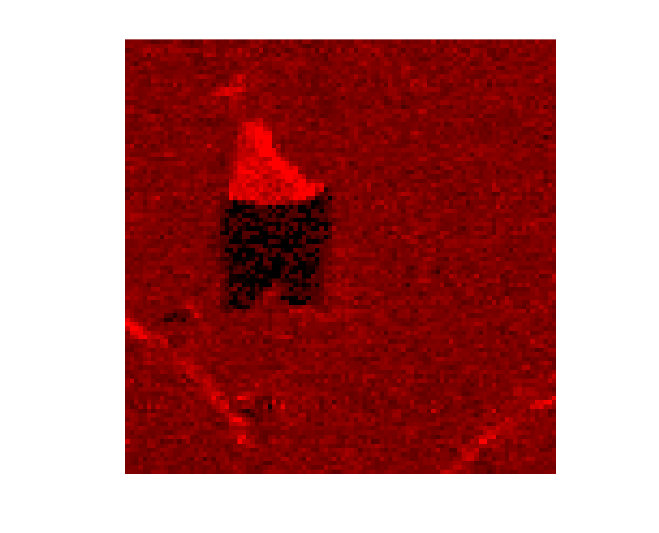

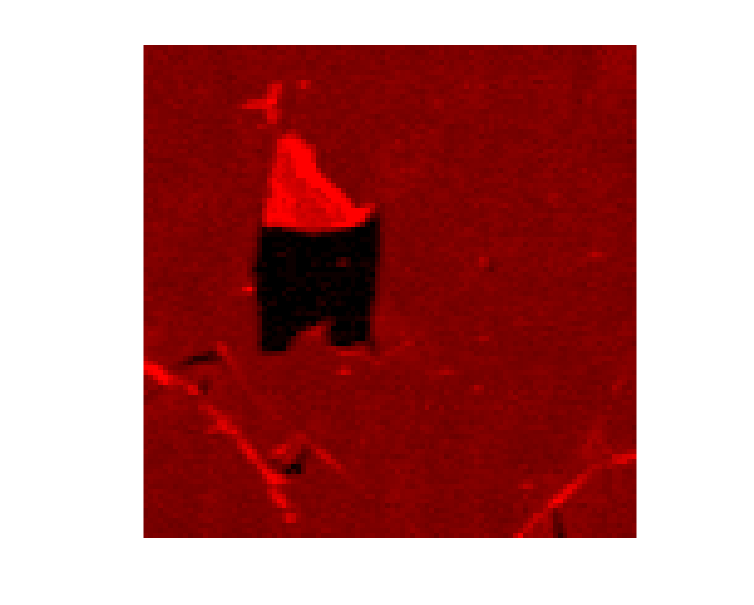

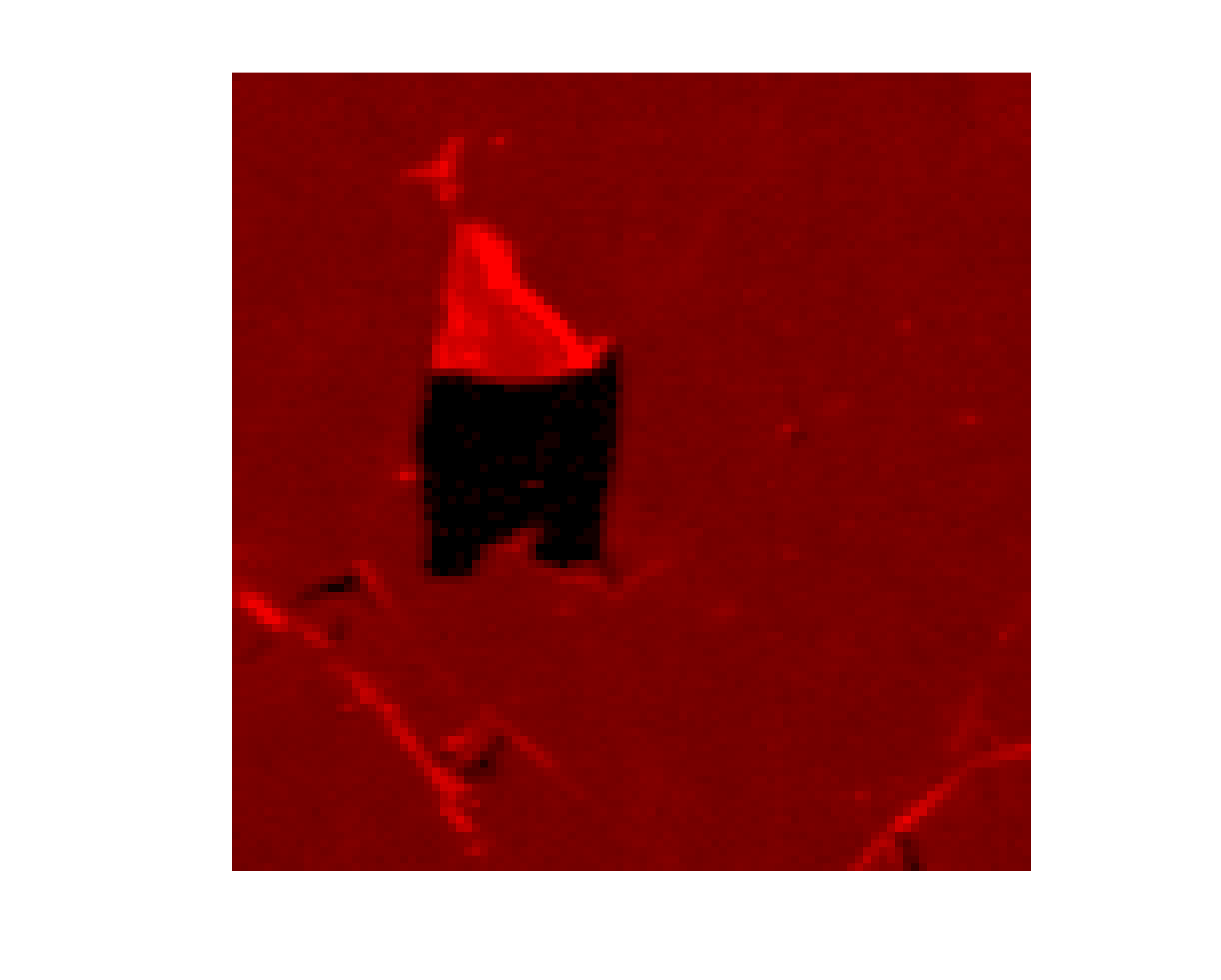

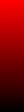


0

150

a.u.


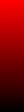


0

1500

a.u.


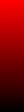


0

5000

a.u.

(a)

(c)

(b)

Supplementary Figure 4: **ai-CRM maps of graphene corresponding to Figure 4 of the main text.** Denoised Raman maps at an integration time of (a) 1 ms, (b) 10 ms and (c) 35 ms. The scale bars correspond to 5 µm.

**Comparison of power averaged scanning rate of graphene oxide and graphene with literatures**


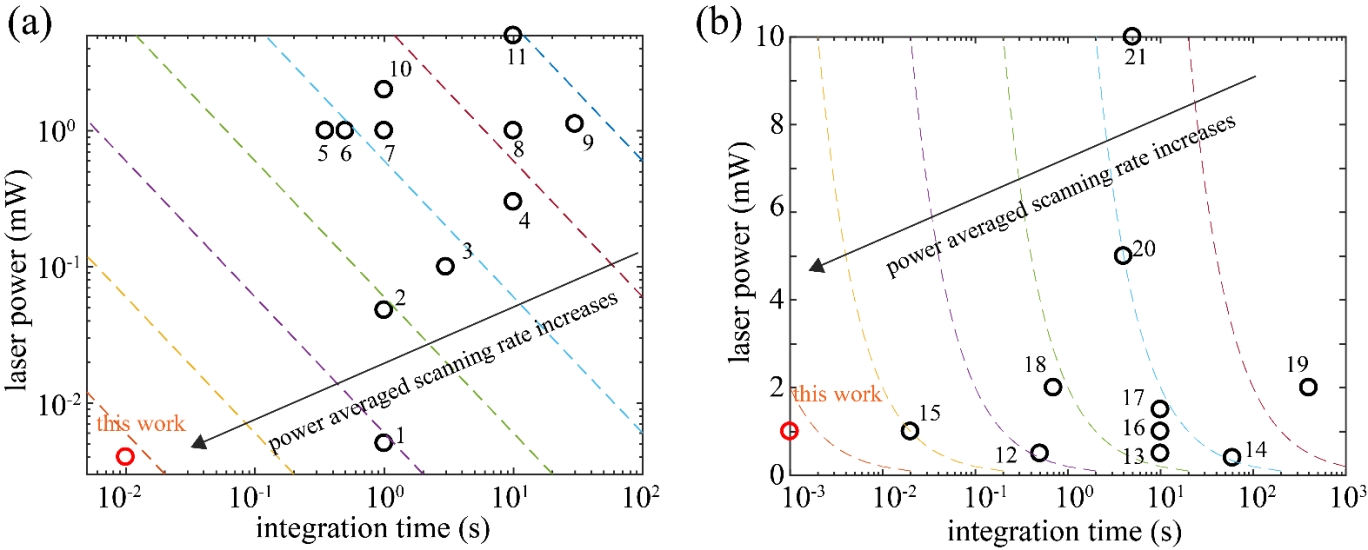


Supplementary Figure 5: **Comparison of our method with previous literatures**. Laser power with integration time used for (a) graphene oxide(*1-11*) and (b) graphene(*12-21*). Note that this is only a rough comparison, because different studies use different instruments, different objectives and operate at different conditions, which all affect the scanning rate. The numbers correspond to the references (see reference list). The gap between neighboring dashed lines represent one order of magnitude difference. Note that the y axis is in log scale in (a) and in linear scale in (b).

**Reduction of GO**

4

0

**
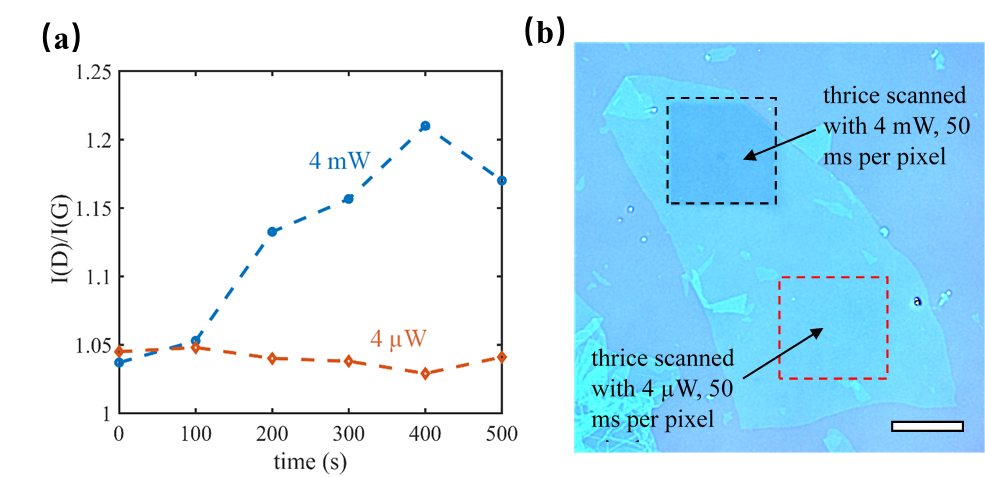
**

Supplementary Figure 6: **Preventing reduction of GO.** (a) Variation of the *I(D)/I(G)* ratio with time corresponding to figure 3(d) and (h) of the main text. Significantly greater change in ratio is observed for the 4 mW case as compared to the 4 µW case, hinting at laser induced reduction. (b) Optical image showing a change in reflection contrast of a GO sheet when mapped thrice with 4mW laser power, whereas no change is observed when scanned with a low laser power of 4 µW. This shows the non-invasive nature of the protocol. Scale bar corresponds to 20 µm.

**Optical and AFM images of exfoliated MoS_2_, WS_2_, and BN**

**
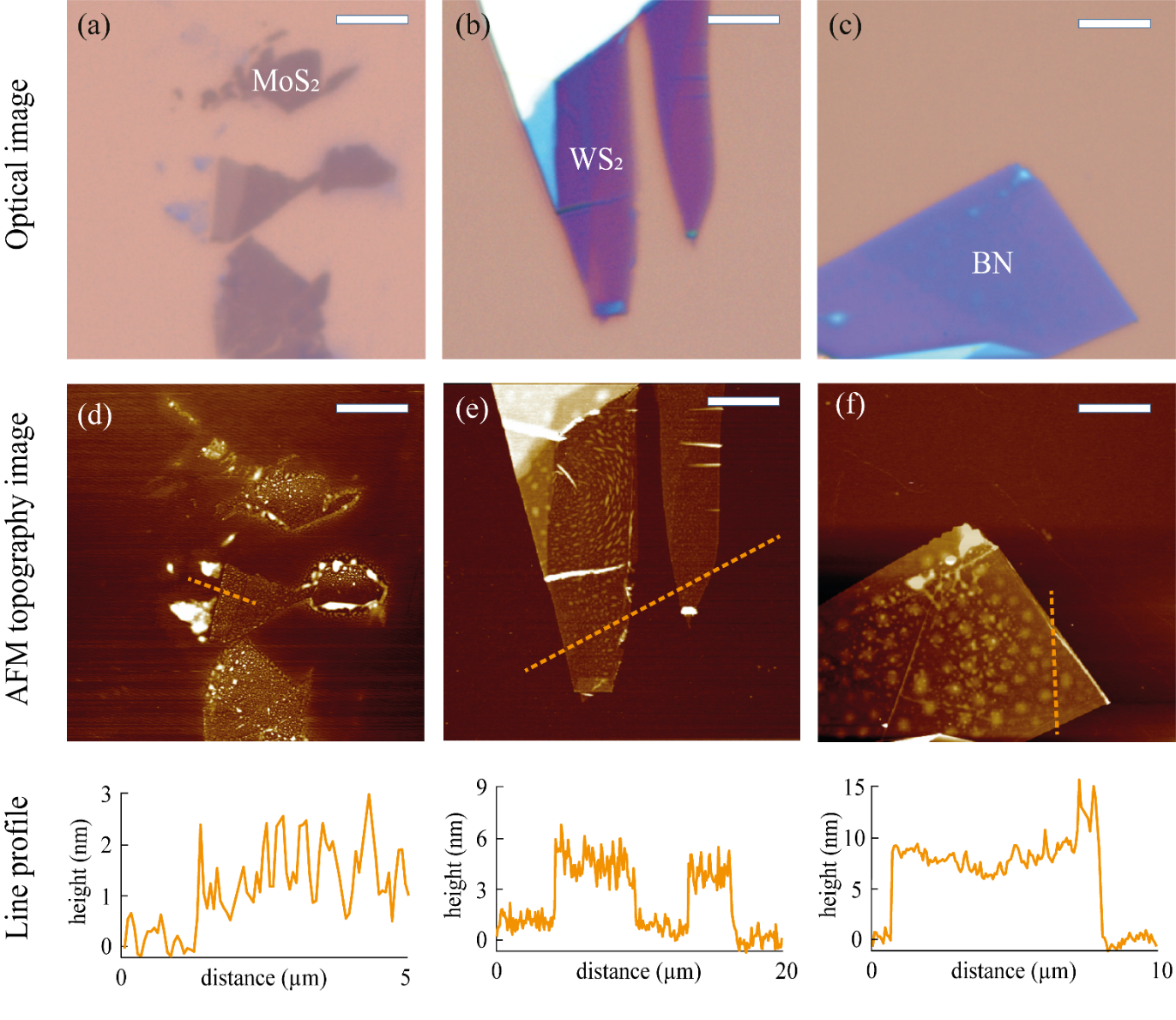
**

Supplementary Figure 7: **Optical and AFM images of MoS_2_, WS2, and BN.** (a), (b) and (c) show the optical images of the mechanically exfoliated MoS_2_, WS_2_ and BN respectively. (d), (e) and (f) show the AFM topography images with the corresponding line profiles (below) of the three materials respectively. Scale bars correspond to 5 µm.

**Raman images of exfoliated MoS_2_, WS_2_, and BN**


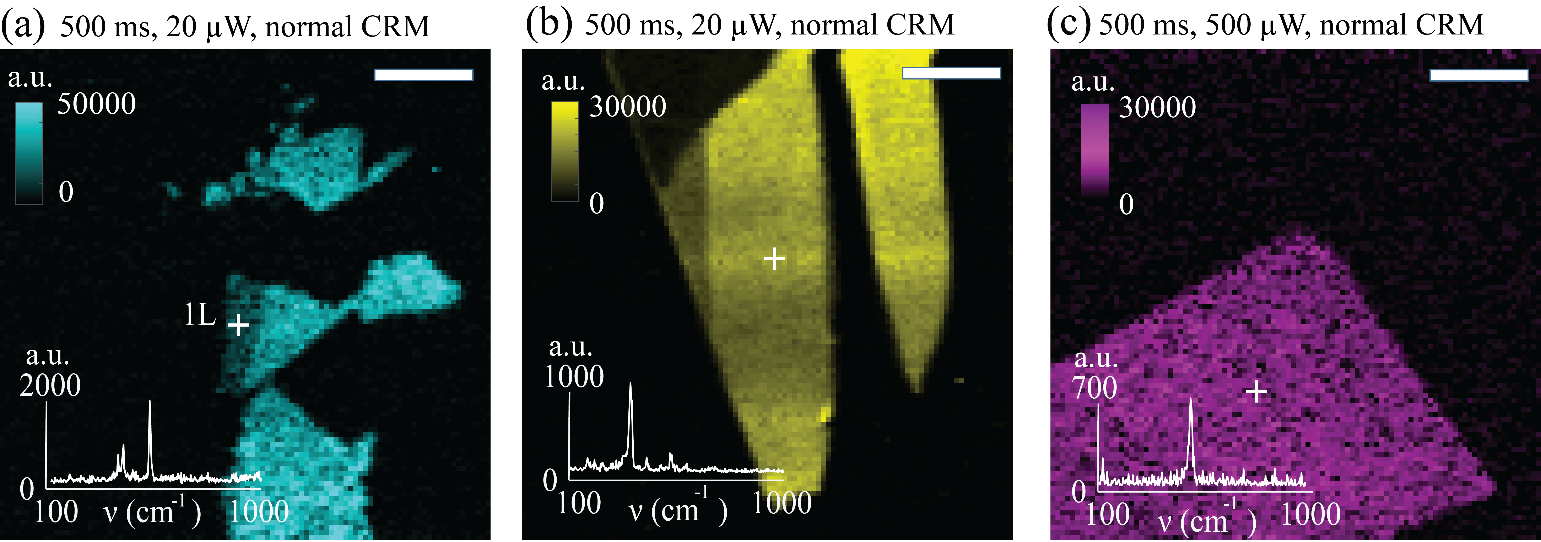


Supplementary Figure 8: **CRM images of MoS_2_, WS_2_, and BN.** (a) Raman mapping of MoS_2_ (b) WS_2_ and (c) BN at 500 ms integration time per pixel. The raw spectra are shown in the inset at the location of the crosshair. The laser power and integration times are mentioned in the figures and scale bars correspond to 5 µm. Resolution of the Raman maps are 100×100 pixels. Scale bars correspond to 5 µm.


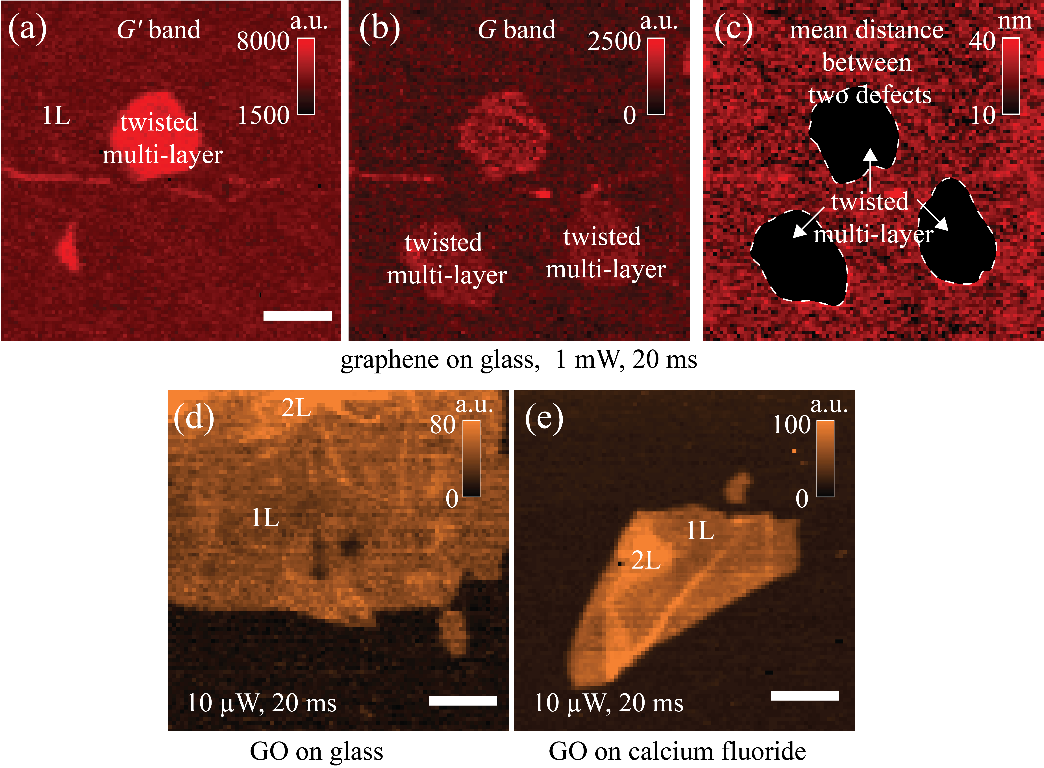


Supplementary Figure 9: **Fast mapping of graphene and GO on arbitrary substrates with ai-CRM** (a) Raman mapping of CVD grown graphene on glass integrated for the *G′* peak, showing the presence of only one multilayer island. (b) Raman mapping of the same region integrated for the *G* peak, showing the presence of all the multilayer islands atop the underlying homogeneous monolayer. The different maps of *G* and *G′* band could be attributed to the presence of a twisted multilayer. (c) Defect density map of same region depicting the mean defect distance around 30 nm. Twisted multilayer regions are not plotted due to the possible complex layer coupling effect. (d) Raman mapping of GO on glass and (e) calcium fluoride. Scale bars correspond to 5 µm.

**Sample MATLAB code**

We encourage readers to test the efficiency of denoising. In the link, a simple sample code and a sample dataset (https://osf.io/d9ucg/?view_only=fbb43fdf5f8d42b5bddfad84442a86d7) are provided. The dataset is in .mat format. Load the dataset in Matlab and then run the code below. Other dataset should be converted to the same format before running the code.

CODE:

%sample matlab code to use PCA for denoising of Raman dataset

%In the sample dataset, RawData is a raw dataset, generated on Witec Raman

%instrument, arranged in a way that the first column represent the

%wavenumbers and the rest columns represent spectra from all pixels (100*100 in this %case).

RawSpectraMatrix=RawData(:, 2:end)';%For PCA, the matrix has to arranged in a way that %each row represent a spectrum

Wavenumbers=RawData(:,1);

%Now apply PCA on the dataset.

mu = mean(RawSpectraMatrix);

[eigenvectors, scores, latent, tsquared, explained]=pca(RawSpectraMatrix);

RawSpectraMatrix=RawSpectraMatrix';

%Select the number of PC to reconstruct dataset, we can first make a Scree

%plot

figure;

semilogx([1:1:size(RawSpectraMatrix,1)],explained,'-o');

%Observe the "knee point" and determine the number of components.

%specify the number of components you want to retain in nComp. Typically it

%is smaller than 10 or around 10. Selecting a few more than needed does not

%significantly undermine the denoising performance.

nComp=[1:12];

%the data after denoising with PCA is denoised_data

DenoisedSpectraMatrix = scores(:,nComp) * eigenvectors(:,nComp)';

DenoisedSpectraMatrix = bsxfun(@plus, DenoisedSpectraMatrix, mu);

DenoisedSpectraMatrix=DenoisedSpectraMatrix';

pixel=500; %Select a random pixel to check the denoising effect

figure;plot(Wavenumbers, DenoisedSpectraMatrix(:,pixel)+250,'linewidth', 1.5);

hold on;

plot(Wavenumbers, RawSpectraMatrix(:,pixel),'linewidth', 1.5);

**References**

1. K. W. Silverstein *et al.*, Voltage-reduced low-defect graphene oxide: a high conductivity, near-zero temperature coefficient of resistance material. *Nanoscale* **11**, 3112-3116 (2019).

2. J. S. Mehta, A. C. Faucett, A. Sharma, J. M. Mativetsky, How Reliable Are Raman Spectroscopy Measurements of Graphene Oxide? *The Journal of Physical Chemistry C* **121**, 16584-16591 (2017).

3. H. Yang, H. Hu, Y. Wang, T. Yu, Rapid and non-destructive identification of graphene oxide thickness using white light contrast spectroscopy. *Carbon* **52**, 528-534 (2013).

4. J. Huang *et al.*, Mechanism of Cellular Uptake of Graphene Oxide Studied by Surface-Enhanced Raman Spectroscopy. *Small* **8**, 2577-2584 (2012).

5. S. Eigler *et al.*, Statistical Raman Microscopy and Atomic Force Microscopy on Heterogeneous Graphene Obtained after Reduction of Graphene Oxide. *The Journal of Physical Chemistry C* **118**, 7698-7704 (2014).

6. D. Kostiuk *et al.*, Reliable determination of the few-layer graphene oxide thickness using Raman spectroscopy. *Journal of Raman Spectroscopy* **47**, 391-394 (2016).

7. P. Vecera *et al.*, Degree of functionalisation dependence of individual Raman intensities in covalent graphene derivatives. *Sci Rep* **7**, 45165 (2017).

8. X. Li *et al.*, Simultaneous Nitrogen Doping and Reduction of Graphene Oxide. *Journal of the American Chemical Society* **131**, 15939-15944 (2009).

9. M. M. MacInnes, S. Hlynchuk, S. Acharya, N. Lehnert, S. Maldonado, Reduction of Graphene Oxide Thin Films by Cobaltocene and Decamethylcobaltocene. *ACS Appl. Mater. Interfaces* **10**, 2004-2015 (2018).

10. H. Wang, J. T. Robinson, X. Li, H. Dai, Solvothermal Reduction of Chemically Exfoliated Graphene Sheets. *Journal of the American Chemical Society* **131**, 9910-9911 (2009).

11. C. Tyagi *et al.*, Evidence of Ion-Beam-Induced Annealing in Graphene Oxide Films Using in Situ X-Ray Diffraction and Spectroscopy Techniques. *The Journal of Physical Chemistry C* **122**, 9632-9640 (2018).

12. H. Nakajima *et al.*, Imaging of local structures affecting electrical transport properties of large graphene sheets by lock-in thermography. *Science Advances* **5**, eaau3407 (2019).

13. S. Barg *et al.*, Mesoscale assembly of chemically modified graphene into complex cellular networks. *Nature Communications* **5**, 4328 (2014).

14. H. Liu *et al.*, Photochemical Reactivity of Graphene. *Journal of the American Chemical Society* **131**, 17099-17101 (2009).

15. S. Wagner *et al.*, Noninvasive Scanning Raman Spectroscopy and Tomography for Graphene Membrane Characterization. *Nano Letters* **17**, 1504-1511 (2017).

16. L. Jiao, L. Zhang, X. Wang, G. Diankov, H. Dai, Narrow graphene nanoribbons from carbon nanotubes. *Nature* **458**, 877 (2009).

17. J. E. Lee, G. Ahn, J. Shim, Y. S. Lee, S. Ryu, Optical separation of mechanical strain from charge doping in graphene. *Nature Communications* **3**, 1024 (2012).

18. L. Zhang *et al.*, Janus graphene from asymmetric two-dimensional chemistry. *Nature Communications* **4**, 1443 (2013).

19. Y. Ito *et al.*, High-Quality Three-Dimensional Nanoporous Graphene. *Angewandte Chemie International Edition* **53**, 4822-4826 (2014).

20. X. Wang, H. Dai, Etching and narrowing of graphene from the edges. *Nature Chemistry* **2**, 661 (2010).

21. L. Jiao, X. Wang, G. Diankov, H. Wang, H. Dai, Facile synthesis of high-quality graphene nanoribbons. *Nature Nanotechnology* **5**, 321 (2010).
